# Association of Polybrominated Diphenyl Ethers (PBDEs) and Polychlorinated Biphenyls (PCBs) with Hyperthyroidism in Domestic Felines, Sentinels for Thyroid Hormone Disruption

**DOI:** 10.1186/s12917-017-1031-6

**Published:** 2017-05-03

**Authors:** Kyla M. Walter, Yan-ping Lin, Philip H. Kass, Birgit Puschner

**Affiliations:** 1 Department of Molecular Biosciences, School of Veterinary Medicine, University of California, 1089 Veterinary Medicine Dr., Davis, CA 95616 USA; 2 Department of Population Health and Reproduction, School of Veterinary Medicine, University of California, 1089 Veterinary Medicine Dr., Davis, CA 95616 USA

**Keywords:** PBDE, PCB, Hyperthyroidism, Feline, Thyroid, Thyroidopathy, Persistent organic pollutants, Endocrine disruption

## Abstract

**Background:**

Hyperthyroidism is the most common endocrine disorder observed in domestic felines; however, its etiology is largely unknown. Two classes of persistent organic pollutants, polybrominated diphenyl ethers (PBDEs) and polychlorinated biphenyls (PCBs) are known to interfere with thyroid hormone (TH) signaling and regulation; thus, it is postulated that they contribute to the etiopathogenesis of feline hyperthyroidism and pose a risk to humans and other species. In this case-control study, the concentrations of 13 PBDE and 11 PCB congeners were measured by gas chromatography mass spectrometry in serum or plasma samples from 20 hyperthyroid and 31 control cats in order to investigate the association between concentration of PBDE and PCB congeners and feline hyperthyroidism. Logistic regression analysis was used to determine whether elevated concentrations of individual congeners were associated with a higher risk of feline hyperthyroidism.

**Results:**

Hyperthyroid cats had higher concentrations of four PBDE congeners (BDE17, BDE100, BDE47, and BDE49) and five PCB congeners (PCB131, PCB153, PCB174, PCB180, and PCB196), compared to control cats. In addition, the sum of both PBDE and PCB congener concentrations were elevated in the hyperthyroid group compared to control cats; however, only the increased PCB concentrations were statistically significant. The sum total PBDE concentrations in our feline samples were approximately 50 times greater than concentrations previously reported in human populations from a geographically similar area, whereas sum total PCB concentrations were comparable to those previously reported in humans.

**Conclusions:**

These observational findings support the hypothesis that PBDEs and PCBs may contribute to the etiopathogenesis of hyperthyroidism in felines. As domestic house cats are often exposed to higher concentrations of PBDEs than humans, they may serve as sentinels for the risk of TH disruption that these pollutants pose to humans and other species.

**Electronic supplementary material:**

The online version of this article (doi:10.1186/s12917-017-1031-6) contains supplementary material, which is available to authorized users.

## Background

Hyperthyroidism is recognized as the most common feline endocrinopathy and is a major cause of morbidity in middle-aged and elderly cats [1]. Feline hyperthyroidism (FH) was first described as a distinct disorder in 1979 [2, 3] and has since increased consistently in prevalence [4, 5]. It is currently estimated to affect over 10% of elderly cats; however, prevalence varies geographically [1, 6]. Feline hyperthyroidism is clinically similar to toxic nodular goiter in humans, both being caused by adenomatous nodular hyperplasia of the thyroid gland [7, 8]. While the pathology of feline hyperthyroidism has been thoroughly characterized [7, 9–13], the etiology of the disorder is largely unknown. Several epidemiologic studies have attempted to identify risk factors associated with FH; however, a single dominant causal factor has not been identified. The scientific literature strongly supports that FH is a complex multifactorial disorder with a significant environmental component [4, 5, 14–18]. As domestic cats and their human owners share their environment, it is probable that environmental factors that contribute to FH may also adversely impact the thyroid health of humans and other animal species.

**Table 1:** Comparison of population demographics and characteristics between feline control and hyperthyroid study groups

Variable	Control (*31*)	Hyperthyroid (*20*)	*p*-value
Age – median (5th–95th percentiles)	12 (3–20)	15 (11–18)	0.040
Gender – percent			
Male	55	45	0.56
Female	45	55	
Total lipids (g/ml) – mean ± SD	0.0057 ± 0.002	0.0059 ± 0.001	0.22
Total T4 (μg/dl) – mean ± SD	2.15 ± 0.68	8.24 ± 4.05	<.001

It has been hypothesized that exposure to thyroid-disrupting chemicals in the environment may cause thyroid gland dysfunction and contribute to the pathogenesis of FH. A number of environmental contaminants disrupt thyroid hormone (TH) signaling and homeostasis at multiple levels of hormone action, including two classes of persistent organic pollutants found in high levels in the environment: polychlorinated biphenyls (PCBs) and polybrominated diphenyl ethers (PBDEs). Given that PCBs and PBDEs are known TH disruptors, environmental exposure to these compounds may contribute to the development of thyroid hyperplasia, leading to hyperthyroidism. Both PCBs and PBDEs have high chemical and thermal stability, making them useful for a number of commercial applications. PCBs were widely used, beginning in the 1920s, as electrical insulators in transformers, capacitors, and heat exchangers, and as stabilizers in paints, plastics, and rubber products [19]. Major production of PBDEs began in the early 1970s, shortly before FH was initially reported, for use as flame retardants in electronics, home furnishings, and foam products, including pet toys and bedding [20–22]. While commercial production of PCBs was banned in 1979 and two commercial formulations of PBDEs (pentaBDE and octaBDE) were banned or phased out of production in some states in the U.S. in 2006, these compounds remain ubiquitous environmental pollutants due to their resistance to degradation and ability to bioaccumulate in human and animal tissues [23, 24]. Previous studies have reported serum PBDE concentrations in cats that are 10–100 fold greater than median levels measured in adult human populations from their respective countries [25–28]. Therefore, domestic cats may serve as sentinels for adverse health consequences associated with exposure to these environmental contaminants.

**Fig. 1:**
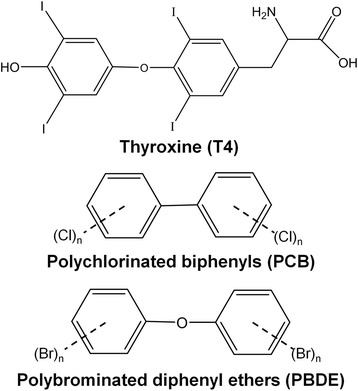
Chemical structures of the thyroid hormone thyroxine (T4), polychlorinated biphenyls (PCBs), and polybrominated diphenyl ethers (PBDEs)

PCBs and PBDEs each consist of a total of 209 congeners with varying degrees of chlorination and bromination, respectively, and the effects of individual congeners and their metabolites on TH signaling and regulation are not well understood. While these compounds may derive some of their toxicity from their structural similarity to thyroid hormones thyroxine (T4) and triiodothyronine (T3) (Fig. 1), a wide range of congeners must be evaluated for potential association with FH. The goal of this study was to investigate whether elevated serum or plasma concentrations of select PBDE and/or PCB congeners are associated with FH to further examine the hypothesis that these persistent pollutants may contribute to the pathogenesis of FH.

## Methods

### Study population

Plasma and serum samples were obtained from client-owned cats visiting the University of California Davis William R. Pritchard Veterinary Medical Teaching Hospital (VMTH) (Davis, CA, USA) during 2012 and 2013 for routine appointments. Samples were stored at −20 °C prior to analysis. Hyperthyroidism was diagnosed on the basis of consistent clinical signs observed by a VMTH clinician and total serum T4 concentrations measured above the reference range of 1.1–3.3 μg/dl. Both newly diagnosed cases of FH and cats receiving treatment for previously diagnosed FH were included in the study; however, all reported T4 values were from the time of diagnosis. Control samples were collected randomly from feline patients presented to the VMTH for routine appointments and determined to be free of FH based on the absence of clinical signs of hyperthyroidism or any other endocrine disease. In total, 20 hyperthyroid cats and 31 control cats were included in the analysis. Written consent was obtained from all owners to permit use of their cats’ samples in the study.

### Extraction of analytes

Samples were stored at −20 °C and thawed on ice prior to extraction. The method used for the extraction of PBDE and PCB analytes and the materials used for extraction have been detailed previously [29]. Briefly, 0.5 ml aliquots of serum or plasma were mixed with 0.5 ml of pure formic acid, ultrasonicated for 10 min, and filtered gravimetrically through Waters Oasis HLB SPE cartridges (Milford, MA, USA), which had been previously conditioned with methanol and ultrapure water with formic acid and methanol (*v*/v/v, 94.5/0.5/5). SepPak cartridges (Sep-pak® Light Silica cartridges, Waters, Milford, MA, USA) were placed beneath SPE columns and analytes were eluted with three washes of 3 ml dichloromethane under vacuum. Eluents were collected in disposable glass tubes containing 100 μL of 1 ng/ml Mirex as an internal standard to evaluate instrument performance. Samples were dried in a water bath (40 °C) using a nitrogen evaporator (Organomation Associates, Inc., Berlin, MA, USA), reconstituted in 100 μl of isooctane, and transferred into auto-sampler vials for analysis. ^13^C_12_ labeled 2,3′,4,4′,5-Penta BDE (^13^C_12−_BDE-118) and ^13^C12 labeled 2,2′,3′,4,5-Pentachlorobiphenyl (13C12-PCB-97) were used as surrogate internal standards throughout the extraction and analytical procedures (Cambridge Isotope Laboratories, Inc., Tewksbury, MA, USA). Nine calibration samples were prepared by adding PBDE and PCB analytical standards (Accustandard, Inc., New Haven, CT, USA) to 0.5 ml of control human serum purchased from Sigma-Aldrich Corp. (St. Louis, MO, USA) to generate the following PBDE and PCB concentrations: 0.04, 0.1, 0.2, 0.8, 2, 4, 10, 20, and 50 ng/ml. Calibration samples were processed following the same extraction method as described for feline serum and plasma samples above.

### Instrumentation and analysis of analytes

Samples extracts were analyzed for BDE-17, −28, −47, −49, −52, −66, −85, −95, −99, −100, −153, −154, and −183, and PCB-91, −95, −131, −135, −136, −153, −174, −175, −176, −180, and −196 by gas chromatography coupled with triple quadruple mass spectrometry (GC/MS/MS, Scion TQ triple quadruple mass spectrometer Bruker, Fremont, CA, USA). The detailed description for sample preparation and GC-MS/MS parameters was reported elsewhere [29]. Calibration curves were weighted 1/x. Two different ranges of the calibration standards were used to quantify analytes to improve the linearity of calibration curves. Initially, all analytes were quantified using a calibration curve including standard concentrations of 0.04–20 ng/ml. Following analysis, analytes in any sample calculated to exceed 20 ng/ml were then quantified using a calibration curve including standard concentrations of 2–50 ng/ml. The lower limit of detection (LOD) and quantification (LOQ) were established based on the lowest calibrator with a signal-to-noise ratio of 3:1 and 10:1, respectively. In order to facilitate the statistical analysis, a non-detected congener was assigned a value of the corresponding LOD divided by ½.

### Quality control

The accuracy was assessed using the certified National Institute of Standards and Technology (NIST) reference standard serum SRM1957 (Gaithersburg, MD, USA) and three quality control (QC) samples of human control serum fortified with all PBDEs and PCBs at concentrations of 0.2, 2 and 10 ng/ml. NIST and QC samples were prepared following the same extraction method as described for the feline samples and analyzed in parallel with each group of feline samples. For each group of samples processed and analyzed, the determined concentration of each PBDE and PCB congener in the QC and NIST samples, as quantified by the standard curves, was required to fall within ±20% of the known concentration of the individual congener for the data to be included in the final analysis. The analytical laboratory participates in the Artic Monitoring and Assessment Program (also known as AMAP Ring Test of Persistent Organic Pollutants in Human Serum) (CTQ 2014) for PBDEs analyses and consistently showed excellent performance for PBDE congener analysis with a Z-score < 2.

### Lipid content determination

Total lipid content was calculated from measurements of total cholesterol (TC) and total triglycerides (TG) in each feline sample using standard enzymatic methods at the UC Davis Health System Department of Pathology and Laboratory Medicine [30, 31]. The TC and TG concentrations were used to calculate the total lipids (TL) for each sample using the following equation: [32].

$$ \mathrm{TL}=\left(2.27\ast \mathrm{TC}\right)+\mathrm{TG}+62.3\left( mg/ dl\right) $$

### Statistical analysis

The concentrations of 13 PBDE and 11 PCB congeners in feline serum and plasma samples were described using the median values and the 10th and 90th percentiles. Logistic regression was used to analyze the association between the presence/absence of feline hyperthyroidism and the serum/plasma concentration of individual PBDE or PCB congeners. The association of age and gender on the odds of hyperthyroidism was investigated using Mann-Whitney and chi-square tests of independence, respectively, to determine whether these variables should be controlled for in the logistic regression models. Lipid concentrations were compared between groups using Student’s two-group *t*-test.

The detection frequency (DFR) for each of the 13 PBDE and 11 PCB congeners assessed was determined by calculating the percent of all measured samples in which the concentration of the congener was above the limit of detection. The sum concentration for PBDE and PCB congeners was calculated for all the congeners measured (ƩPBDEs and ƩPCBs) and also for those with DFRs of greater than 40% (ƩPBDE40 and ƩPCB40) in order to investigate both the sum of all selected congeners and the sum of those congeners that were more consistently measured without influence from the less frequently detected congeners. In our discussion, ƩPBDEs and ƩPCBs were compared to previously reported measurements of PBDEs and PCBs in a human population from a geographically similar area as part of a study conducted by the National Health and Nutrition Examination Survey [33].

For each PBDE and PCB congener, a logistic regression model, including congener concentration and age as continuous (linear) variables, was used to analyze the association of congener concentration with odds of hyperthyroidism while controlling for the influence of age. This model allowed statistically adjusting for age as a confounder, so that the adjusted odds ratios reflect the association strictly of the congener, rather than the associations of both congener and age, on hyperthyroid status. Results are reported as odds ratios and 95% confidence intervals (CI). Due to large differences in the median concentrations of different PBDE and PCB congeners, the odds ratios (OR) for congeners with a median serum concentration above 100 ng/g lipid in the hyperthyroid group are presented corresponding to a 100 ng/g lipid increase in congener concentration, and the odds ratios for all remaining congeners are presented corresponding to a 10 ng/g lipid increase in congener concentration. The initial statistical analysis was done using logistic regression in STATA data analysis and statistical software (Stata IC/13, StataCorp LP, College Station, TX, USA) which uses the standard maximum-likelihood-based estimator. Due to the small sample size, any congeners which yielded *p*-values less than 0.10 were subsequently analyzed by exact logistic regression using LogXact statistical software (Cytel Software Corporation, Cambridge, MA, USA).

## Results

### Study population characteristics

The demographic characteristics of the hyperthyroid and control feline study populations are summarized in Table 1. A total of 20 hyperthyroid and 31 control cats were included in the study. The median age of hyperthyroid cats was 15 years (5th – 95th percentile, 11–18 years), compared to a median of 12 years (5th – 95th percentile, 3–20 years) in the control group. A Mann-Whitney rank sum test indicated that age varied significantly between study groups (*p* = 0.040); therefore, age was controlled for in subsequent logistic regression analyses to assess the influence of PBDE and PCB congener concentration on the risk of hyperthyroidism. The percentage of males and females in the control and hyperthyroid groups was not significantly different, (χ^2^ (1) = 0.33, *p* = 0.56), thus it was not included in the logistic regression analyses.

### PBDE and PCB concentrations

The serum/plasma concentrations of 13 PBDE and 11 PCB congeners were determined and normalized to the total lipid concentration for each feline sample. The total lipids did not vary significantly between the hyperthyroid and control groups (*t* (49) = −0.71, *p* = 0.48). Of the 13 PBDE congeners measured, the following 6 had detection frequencies (DFRs) of greater than 40%: BDE 47, BDE 95, BDE 99, BDE 100, BDE 153, and BDE 154. Of the 11 PCB congeners measured, the following five congeners had DFRs of greater than 40%: PCB 131, PCB 153, PCB 174, PCB 176, and PCB196. The sum of concentrations for PBDE and PCB congeners in all samples, the control, and the hyperthyroid group was calculated for all the congeners measured (ƩPBDEs and ƩPCBs) and also for those with DFRs of greater than 40% (ƩPBDE40 and ƩPCB40) (Additional file 1: Table S1). The ƩPBDE40 and ƩPCB40 concentration values were used to assess the association of total PBDE and PCB concentration with feline hyperthyroidism.

**Fig. 2:**
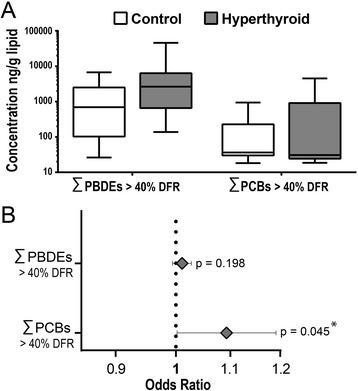
Distribution of ƩPBDEs and ƩPCBs concentrations in feline samples and the odds ratios of feline hyperthyroidism associated with a 100 ng/g lipid increase in ƩPBDEs and ƩPCBs. **a** Box and whiskers diagrams of the ƩPBDEs and ƩPCBs concentrations shows the distribution of measured concentrations (ng/g lipid) for control (*white boxes*) and hyperthyroid (*gray boxes*) feline samples. Only congeners with a detection frequency (DFR) greater than 40% were included in the sum value. The upper and lower boundaries of the boxes represent the 75th and 25th percentiles, respectively, and the line within the boxes denotes the median value. The *upper* whiskers show the 90th and the *lower* whiskers the 10th percentile. **b** Odds ratios were determined by logistic regression analysis of the relationship between ƩPBDEs and ƩPCBs and feline hyperthyroidism, controlling for age of feline study participants. *Error bars* represent the 95% confidence interval. An odds ratio that is significantly greater than one indicates that an increase in the serum concentration of the specific congener is associated with increased odds of having feline hyperthyroidism. *p ≤ 0.05 in exact logistic regression controlling for age of feline study participants

The ƩPBDE40 had mean (±SE) and median (10–90 percentiles) concentrations of 4692 ng/g lipid (± 1.467 ng/g lipid) and 812 ng/g lipid (38.3–8723 ng/g lipid), respectively. The ƩPCB40 had mean (±SE) and median (10–90 percentile) concentrations of 497 ng/g lipid (± 158 ng/g lipid) and 34.5 ng/g lipid (18.5–1251 ng/g lipid), respectively (Additional file 1: Table S1). The distribution of ƩPBDE40 and ƩPCB40 concentrations for the hyperthyroid and control feline samples are shown in Fig. 2a and given as a complete data set (Additional file 1: Table S1). The mean and median values of the ƩPBDEs were higher in the hyperthyroid group of feline samples compared to the control group. For the ƩPCBs, the mean values were higher in the hyperthyroid group; however, the medians were similar. Logistic regression analysis was used to examine the association of the ƩPBDE40 or ƩPCB40 concentrations with the occurrence of feline hyperthyroidism, while controlling for the influence of age. An odds ratio greater than one indicates that there is an association between an increase in the ƩPBDEs or ƩPCBs concentrations and the incidence of feline hyperthyroidism. The odds ratios for a 100 ng/g lipid increase in ƩPBDE40 or ƩPCB40 (Fig. 2b) were 1.000108 and 1.000885, respectively. However, only the odds ratio for ƩPCB40 was statistically significant (*p* < 0.05), indicating that an increase in the serum or plasma concentration of ƩPCB40 is associated with the occurrence of feline hyperthyroidism.

**Fig. 3:**
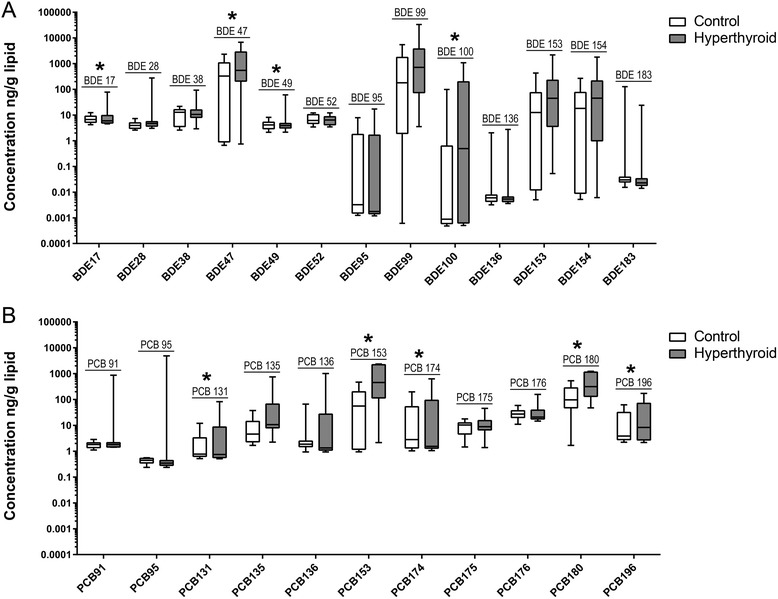
Box and whiskers diagram of PBDE and PCB congener concentrations in feline samples. The distribution of measured concentrations of each PBDE **a** and PCB **b** congener are given as lipid normalized values (ng/g total lipids) for control (*white boxes*) and hyperthyroid (*gray boxes*) feline samples. The *upper* and *lower* boundaries of the boxes represent the 75th and 25th percentiles, respectively, and the line within the boxes denotes the median value. The upper whiskers show the 90th and the lower whiskers the 10th percentile. *p ≤ 0.05 in exact logistic regression controlling for age of feline study participants

The distribution of individual PBDE and PCB congener concentrations measured (ng/g lipid) in the hyperthyroid and control groups are shown in Fig. 3. The two most abundant PBDE congeners were BDE 47 and BDE 99 (Fig. 3a). BDE 47 was detected in 78% of the feline samples with a median concentration (10-90th percentiles) of 497 ng/g lipid (0.7–4300 ng/g lipid) (Additional file 1: Table S1). BDE 99 was detected in 82% of the feline samples with a median concentration (10-90th percentiles) of 570 ng/g lipid (0.0–5890 ng/g lipid). The median values of BDE 47 and BDE 99 were higher in the hyperthyroid group of feline samples compared to the control group. The median (10–90 percentiles) values of BDE47 were 328 ng/g lipid (0.8–1659 ng/g lipid) for the control group compared to 698 ng/g lipid (0.7–6806 ng/g lipid) for the hyperthyroid group. The median (10–90 percentiles) values of BDE99 were 179 ng/g lipid (0.0–3807 ng/g lipid) for the control group and 995 ng/g lipid (17.8–31,908 ng/g lipid) for the hyperthyroid group. The two most abundant PCB congeners were PCB 153 and PCB 180 (Fig. 3b). PCB153 was detected in 71% of the feline samples with a median concentration (10-90th percentiles) of 78 ng/g lipid (1.2–1846 ng/g lipid) and PCB 180 was detected in 90% of the feline samples with a median (10-90th percentiles) of 175 ng/g lipid (26.5–909 ng/g lipid). The median values of PCB153 and PCB180 were higher in the hyperthyroid group of feline samples compared to the control group. The median (10–90 percentiles) values of PCB153 were 55 ng/g lipid (1.0–468 ng/g lipid) for the control group compared to 1042 ng/g lipid (2.2–2370 ng/g lipid) for the hyperthyroid group. The median (10–90 percentiles) values of PCB180 were 96 ng/g lipid (1.8–481 ng/g lipid) for the control group and 653 ng/g lipid (47.6–1229 ng/g lipid) for the hyperthyroid group. The median (10–90 percentiles), mean (± SE), and DFR for each congener in all feline samples as well as the hyperthyroid and control groups are given as a complete data set (Additional file 1: Table S1).

**Fig. 4:**
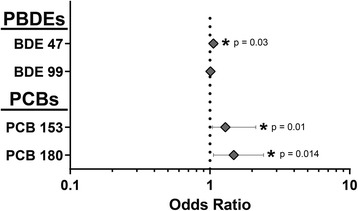
Odds ratios and 95% CI of feline hyperthyroidism associated with a 100 ng/g lipid increase in serum concentration of the four most abundant PBDE and PCB congeners. Odds ratios were determined by logistic regression analysis of the relationship between serum concentrations of individual PBDE or PCB congeners and feline hyperthyroidism, controlling for age of feline study participants. *Error bars* represent the 95% confidence interval. An odds ratio that is significantly greater than one indicates that an increase in the serum concentration of the specific congener is associated with increased odds of having feline hyperthyroidism. *indicates a *p*-value ≤0.050

Logistic regression analysis was utilized to determine odds ratios for each PBDE and PCB congener, describing the relationship between an elevated concentration of a congener and the associated change in the odds of a feline patient having hyperthyroidism. For clarity of data interpretation, the odds ratios of the four most abundant congeners, BDE47, BDE99, PCB153, and PCB180, are presented corresponding to a 100 ng/g lipid increase in serum or plasma concentration as each of these congeners had a median greater than 100 ng/g lipid for the hyperthyroid group (Fig. 4). The odds ratios, 95% confidence intervals and associated *p*-values obtained from logistic regression analysis are shown in Fig. 4 and provided as a complete data set (Additional file 1: Table S2). Of the four congeners measured in highest abundance, three of them, BDE47, PCB153, and PCB180, had positive odds ratios that were significantly greater than 1.0 (*p* < 0.05), indicating that these associations were unlikely to be due to chance in the absence of bias and correctness of model specification. Specifically, the odds ratios for BDE47, PCB153, and PCB180 were 1.06 [95% confidence interval (CI), 1.00–1.13, *p* = 0.030], 1.29 (95% CI, 1.04–2.13, *p* = 0.010), and 1.48 (95% CI, 1.06–2.42, *p* = 0.014), respectively (Fig. 4). These odds ratios indicate that a 100 ng/g lipid increase in the serum concentration of BDE47, PCB153, or PCB180 is associated with a 6.0%, 29%, and 48% increase, respectively, in the odds of feline hyperthyroidism.

**Fig. 5:**
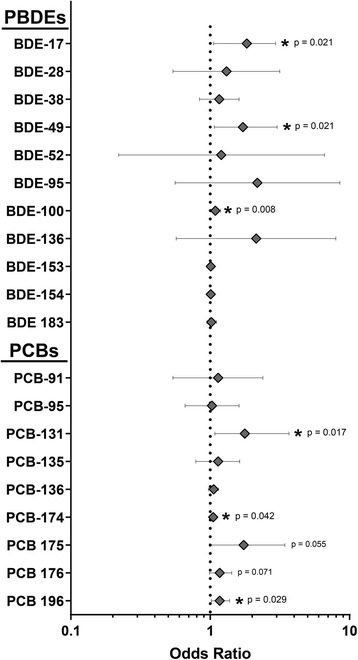
Odds ratios and 95% CI of feline hyperthyroidism associated with a 10 ng/g lipid increase in serum concentration of PBDE and PCB congeners. Odds ratios were determined by logistic regression analysis of the relationship between serum concentrations of individual PBDE or PCB congeners and feline hyperthyroidism, controlling for age of feline study participants. Error bars represent the 95% confidence interval. An odds ratio that is significantly greater than one indicates that an increase in the serum concentration of the specific congener is associated with increased odds of having feline hyperthyroidism. *indicates a *p*-value ≤0.050

The less abundant PBDE and PCB congeners all had median values below 100 ng/g lipid in the hyperthyroid group, and their odds ratios are presented as the increase in odds of hyperthyroidism associated with a 10 ng/g lipid increase in the serum concentration. The odds ratios, 95% confidence intervals, and *p*-values obtained from logistic regression analysis are shown in Fig. 5 and given as a complete data set (Additional file 1: Table S3). Among these less abundant congeners, six additional BDEs and PCBs had positive odds ratios that were significantly greater than 1.0 (*p* < 0.05): BDE17, BDE49, BDE100, PCB131, PCBE174, and PCB196. The odds ratio for a 10 ng/g lipid increase in serum concentration was 1.83 (95% CI, 1.06–2.95, *p* = 0.021) for BDE17, 1.72 (95% CI, 1.07–3.02, *p* = 0.021) for BDE49, 1.09 (95% CI, 1.01–1.19, *p* = 0.008) for BDE100, 1.77 (95% CI,1.08–3.68, *p* = 0.017) for PCB131, 1.05 (95% CI, 1.00–1.11, *p* = 0.042) for PCB174, and 1.17 (95% CI, 1.02–1.38, *p* = 0.029) for PCB196. These odds ratios indicate that a 10 ng/g lipid increase in the serum concentrations of BDE17, BDE49, BDE100, PCB131, PCBE174, or PCB196 is associated with an 83%, 72%, 9.0%, 77%, 5.0%, or 17% increase, respectively, in the odds of a feline patient having hyperthyroidism.

## Discussion

In this study, the serum or plasma concentrations of 13 PBDE and 11 PCB congeners were determined for 20 hyperthyroid and 31 control cats presented to the UC Davis VMTH. PBDEs and PCBs were detected in all samples, with BDE47, BDE99, PCB153, and PCB180 having the highest mean and median concentrations of all the congeners. An exact logistic regression model was utilized to determine whether the concentration of individual congeners or the sum of congeners was correlated with the incidence of feline hyperthyroidism (FH). Exact logistic regression analysis indicated that increased concentrations of BDE17, BDE47, BDE49, BDE100, PCB131, PCB153, PCB174, PCB180, and PCB196 were associated with diagnosis of feline hyperthyroidism. In addition, the sum concentration of PCB congeners with DFRs greater than 40% (ƩPCB40) was also correlated with incidence of FH.

Feline hyperthyroidism has been thoroughly characterized and typically results from autonomous secretion of excess THs from benign adenomas in the thyroid follicles [9, 34]. However, the etiology of the disease and the pathological development of thyroid adenomatous hyperplastic cells are not well understood. A combination of immune, genetic, nutritional, and environmental factors have been hypothesized and studied; however, no single dominant etiological factor has been identified. Studies suggest that FH has a significant environmental contribution, and exposure to thyroid-disrupting compounds, such as PBDEs and PCBs, has been hypothesized as a likely contributing factor [1, 18]. Both indoor lifestyle and consumption of canned food have been linked to FH, and each of these may contribute to the total body burden of PBDEs and PCBs. The levels of PBDEs in dust from the homes of hyperthyroid cats was significantly higher than in dust from the homes of age-matched euthyroid control cats [35], and the PBDE congener pattern was similar in the serum of house cats and house dust samples [27], indicating that house dust may be a dominant source of PBDE exposure. In addition, PBDEs, PCBs, and some of their metabolites have been detected in canned pet food and may be a source of exposure [36]. Considering the association of FH with indoor lifestyle and consumption of canned food, it is important to keep in mind that elevated BPDE and PCB exposure may merely be associated with indoor lifestyle and/or canned food consumption and that another associated factor is the true causal mechanism of hyperthyroidism.

It has been a longstanding hypothesis that PBDEs and PCBs contribute to the development of FH; however, previous studies of the relationship between serum or plasma concentrations of PBDE and/or PCB congeners and hyperthyroidism have generated variable results. While some found no evidence linking PBDE or PCB profiles to FH [25, 27], a few have reported indirect evidence linking PBDE or PCB exposure to FH despite not identifying differences in congener concentrations between hyperthyroid and control cats. These include elevated PBDEs in dust from houses containing hyperthyroid cats [35] and altered ratios of BDE100/BDE99 in hyperthyroid cats [26]. Recently, Norrgran et al. [39] identified three BDE congeners (BDE99, BDE153, and BDE183) and one PCB congener (PCB153) that were significantly higher in hyperthyroid cats than control cats in Sweden. There are a number of factors that may account for the variability in previously reported results and may confound future studies. First, the concentrations of PBDE and PCB congeners show high variability within groups and most studies are limited by small sample size. Both of these factors can greatly limit the power of statistical analyses. In our study, an exact logistic regression analysis was utilized to analyze the correlation of congener concentrations to feline hyperthyroidism in order to prevent small-sample size bias that may occur as a result of maximum likelihood estimation of the normal logistic regression model. In addition, the exact logistic regression does not assume normality or equal variance. Thus, exact logistic regression analysis of PBDE and PCB congener concentrations and their relationship to FH provides a robust analysis for these data. The individual PBDE and PCB congeners that are measured and reported also vary by study, thus the ƩPBDE and ƩPCB values reported are not consistent between studies. While it is common practice to report data only for congeners above a certain percent detection frequency, this biases the analysis toward high abundance congeners. This approach may prevent the identification of low abundance congeners with TH-disrupting capability. Thus, it is important to assess the potential impact of all congeners detected and analyze the total PBDE and PCB congeners as well as specific groups of high abundance congeners that are routinely measured. This would improve overall comparability between studies. In addition, the PBDE and PCB profiles vary regionally, depending on the technical mixtures that have been most prominently used, thus congener collinearity also varies. In our study, as well as several human studies conducted in the U.S.A., BDE-47, −99, −100, −153, and −154 were found in greatest concentration and are the primary components of the penta-BDE mixture DE71, [37] a product widely used in North America to treat polyurethane foam in furniture to comply with California Technical Bulletin 117. In contrast, the most prevalent BDE congeners in feline serum samples in Sweden were BDE209 and BDE207 with BDE99 accounting for only 13% total body burden, compared to 44% in a U.S. population [28]. Therefore, regional variability in PBDE congener profile will likely influence the collinearity of congeners in human or feline samples, thus different congeners may be identified in different populations as being associated with FH. For PCBs, the congener profiles may be more consistent, as PCB153 and PCB180 have been identified as the most prevalent congeners in human serum [38], feline serum [28], and in our study. However, variability in the congener profiles may still contribute to inter-study variation.

PBDEs and PCBs each consist of 209 individual congeners with slightly different structural conformations and varying propensities to interfere with TH signaling and feedback; therefore, we have examined the structure of the congeners elevated in hyperthyroid feline samples to assess potential structural patterns indicative of TH disruption. Among these congeners (BDE17, BDE47, BDE49, BDE100, PCB131, PCB153, PCB174, PCB180, and PCB196), all have bromine or chlorine molecules located at the 2, 2′, and 4 positions on their benzene rings. Halogens in these positions create a structural pattern similar to the three iodine molecules in the most active form of TH, triiodothyronine (T3). Among the remaining fourteen congeners that were not significantly elevated in the hyperthyroid group, six had the same pattern of halogenation. However, two of these congeners (PCB175 and PCB176) had *p*-values that were near significant (0.055 and 0.071, respectively) and another three (BDE99, BDE153, and BDE183) were previously identified as being elevated in a group of hyperthyroid cats [39]. It is possible that a study with a larger sample size may show an association between these congeners and feline hyperthyroidism; however, a more robust structure-activity relationship analysis would be necessary to determine whether this halogenation pattern contributes to the ability of specific PBDE and PCB congeners to interfere with TH function. This information may be extremely useful in identifying congeners that pose the greatest risk for TH disruption and thyroid disease in both feline and human populations.

PBDEs and PCBs have been implicated as TH disruptors in both epidemiologic and laboratory studies. However, the relationship between PBDE exposure and circulating TH levels is poorly understood, with many human epidemiological studies presenting conflicting results [40–45], demonstrating both positive and negative correlations with TH and TSH levels. A meta-analysis of 16 epidemiologic studies recently concluded that the relationship between PBDE exposure and thyroid function is dependent on the serum PBDE concentrations; there was a negative correlation with TSH when median PBDE levels were <30 ng/g lipid and a positive correlation when PBDE levels were >100 ng/g lipid [46]. This proposed u-shaped response curve suggests that multiple mechanisms may contribute to the disruption of circulating thyroid hormones that are likely dependent on concentration and duration of exposure. Human epidemiologic studies on PCB exposure have demonstrated inverse correlations between exposure and circulating THs in some but not all studies conducted [47–53].

While circulating levels of T4, T3, and TSH have been classically used to evaluate endocrine disrupting potential of PBDEs, PCBs, and other compounds, these endpoints may not always predict a compound’s TH-disrupting potential at a cellular level. In PCB-exposed rodent populations with decreased circulating T4, upregulation of the TH-responsive gene RC3 was observed in the cerebral cortex [54], highlighting the importance of evaluating the cellular mechanisms of PBDE- and PCB-induced TH-disruption. TH disruption can occur at multiple levels of hormone action including TH synthesis and release, transport through circulation and across membranes, receptor mediated gene expression, metabolism and clearance, and feedback in the pituitary [55, 56], thus PBDE-and PCB-induced TH disruption may occur through multiple mechanisms. Individual PBDE and/or PCB congeners and their metabolites have demonstrated disrupting potential through the following mechanisms: competitive binding to TH serum transport protein transthyretin (TTR), induction of glucuronidation and sulfation enzymes, interference with TH membrane transporters or intracellular deiodinases, and direct interference with the TH receptor (TR) complex [57–65]. There are significant gaps in the understanding of how each of these mechanisms contributes to chronic PBDE or PCB exposure and resulting TH disruption.

Previous studies, conducted on feline populations in Sweden, Australia, and multiple locations within the U.S.A., have measured serum PBDE concentrations 10–100 fold greater than median levels in adult human populations from their respective countries. In contrast, the measured concentrations of PCBs in feline serum samples are similar to those observed in human populations. The population evaluated in this study spans many cities in Northern California, USA, and the concentrations of individual PBDE and PCB congeners as well as the sum of congeners, ƩPBDEs and ƩPCBS, measured are within a similar range as those previously measured in a population of house cats from California’s San Francisco Bay Area. As previously discussed by Guo et al. [27], the levels of PBDEs that have been observed in California house cats, are approximately 50 times higher than in California human residents sampled in 2003 and 2004 in the National Health and Nutrition Examination Survey, which are among the highest in the world [33]. Cats are likely to have an increased body burden of PBDEs because of their reduced capacity to metabolize these compounds via glucuronidation and increased exposure to PBDE-containing furniture, textiles and house dust [27, 66]. It is possible that chronic exposure to these contaminants at the high levels observed in feline circulation may lead to the development of autonomously secreting hyperplastic cells through multiple mechanisms of action, leading to hyperthyroidism.

With highly elevated exposure to PBDEs and similar exposure to PCBs, house cats may experience the effects of exposure to these pollutants more than humans and other species with lower contaminant levels. Thus, these highly exposed populations of domestic cats can serve as sentinels for the adverse health effects that may occur following prolonged elevated exposure to these persistent organic pollutants. FH is pathologically similar to the human condition of toxic nodular goiter, as both conditions are characterized by the excessive production of THs from thyroid follicles which is autonomous from TSH stimulation [7]. This condition has only been observed in humans and cats; therefore, FH may serve as a unique model for the study of human toxic nodular goiter and its risk factors. The identification of PBDE and PCB congeners associated with thyroid disease among cats is also important within the broader field of environmental health, due to the important roles that TH plays during early development and throughout life. Disruption of TH signaling during critical stages is likely to result in abnormalities in neurodevelopment, growth, energy balance, and reproduction [67]. Therefore, it is essential to develop a broader understanding of the risk that PBDE and PCB exposure poses to the health of both human and animal populations.

## Conclusions

This study demonstrates that elevated exposure to both PBDE and PCB congeners is associated with FH, supporting the hypothesis that these persistent organic pollutants may contribute to the etiopathogenesis of feline hyperthyroidism and suggests that they may have adverse impacts on thyroid health in humans and other animal species. However, the power of our study to detect possible associations with other congeners and potential risk factors, as well as possible departures from linearity between congener concentrations and the log odds of hyperthyroidism, may have been limited by the small sample size and being unable to obtain additional epidemiological data corresponding to the feline patients included in the study. To develop a more thorough understanding of the role that PBDE and PCB body burden plays in the pathogenesis of FH, longitudinal studies to determine the links between exposures to PBDEs and PCBs and development of FH from early life to elderly felines are needed. Thyroid hormone function in the body is critical for proper execution of many developmental processes and the function of most organ systems throughout life across many species, thus there are many adverse implications of TH disruption. The current and future progress among this field of study will greatly contribute not only to our understanding of feline hyperthyroidism, but also to human toxic nodular goiter, and the adverse impacts of chemically-induced TH-disruption that may affect human and animal health spanning all life stages.

## Additional file

Additional file 1: Table S1. Distribution of PBDE and PCB congener concentrations measured in serum and plasma samples obtained from hyperthyroid and control feline patients. **Table S2.** Odds ratios of feline hyperthyroidism by serum concentrations of the four most abundant PBDE and PCB congeners. **Table S3.** Odds ratios of feline hyperthyroidism by serum concentrations of PBDE and PCB congeners. (DOCX 20 kb)

## Abbreviations

FH: Feline hyperthyroidism
PBDE: Polybrominated diphenyl ether
PCB: Polychlorinated biphenyl
TH: Thyroid hormone
