# Supplementary material for: Association of Polybrominated Diphenyl Ethers (PBDEs) and Polychlorinated Biphenyls (PCBs) with Hyperthyroidism in Domestic Felines, Sentinels for Thyroid Hormone Disruption
Source: BMC Vet Res. 2017 May 3;13:120. doi: 10.1186/s12917-017-1031-6 (PMC5415813; doi:10.1186/s12917-017-1031-6)
Supplement: Additional file 1: Table S1. — Distribution of PBDE and PCB congener concentrations measured in serum and plasma samples obtained from hyperthyroid and control feline patients. Table S2. Odds ratios of feline hyperthyroidism by serum concentrations of the four most abundant PBDE and PCB congeners. Table S3. Odds ratios of feline hyperthyroidism by serum concentrations of PBDE and PCB congeners. (DOCX 20 kb) [file 12917_2017_1031_MOESM1_ESM.docx]

Additional File 1

Association between Polybrominated Diphenyl Ethers (PBDEs) and Polychlorinated Biphenyls (PCBs) and Hyperthyroidism in Domestic Felines, a sentinel for thyroid hormone disruption

Kyla M. Walter; Yan-ping Lin, PhD; Philip H. Kass, DVM, PhD; Birgit Puschner, DVM, PhD

Contents:

Table S1: Distribution of PBDE and PCB congener concentrations measured in serum and plasma samples obtained from hyperthyroid and control feline patients.

Table S2: Odds ratios of feline hyperthyroidism by serum concentrations of the four most abundant PBDE and PCB congeners.

Table S3: Odds ratios of feline hyperthyroidism by serum concentrations of PBDE and PCB congeners.

**Table S1.** Distribution of PBDE and PCB congener concentrations measured in serum and plasma samples obtained from hyperthyroid and control feline patients.

|  | % DFR | All samples (n=51) | Control (n = 31) | Hyperthyroid (n = 20) |
| --- | --- | --- | --- | --- |
| **PBDEs** |  |  |  |  |
| BDE-17  Mean ± SE  Median (10-90 pctl) | 19% | 11.9 ± 2.4  6.4 (4.5 – 12.8) | 7.5 ± 0.5  6.7 (4.3 – 12.4) | 18.6 ± 6.0  6.0 (4.6 – 71.5) |
| BDE-28  Mean ± SE  Median (10-90 pctl) | 20% | 30.9 ± 12.9  4.0 (2.7 – 121) | 4.4 ± 0.4  4.0 (2.6 – 7.0) | 60.3 ± 26.0  4.7 (3.1 – 281) |
| BDE-38  Mean ± SE  Median (10-90 pctl) | 19% | 15.6 ± 2.6  11.0 (2.9 – 38.1) | 13.3 ± 2.6  12.8 (2.7 – 20.9) | 19.2 ± 5.4  10.6 (3.0 – 54.2) |
| BDE-47  Mean ± SE  Median (10-90 pctl) | 78% | 1,300 ± 294  497 (0.7 – 4,300) | 665 ± 175  328 (0.8 – 1,659) | 2,002 ± 551  698 (0.7 – 6,806) |
| BDE-49  Mean ± SE  Median (10-90 pctl) | 21% | 9.0 ± 2.2  4.0 (2.2 – 17.5) | 5.1 ± 0.8  4.1 (2.2 – 6.7) | 15.0 ± 5.4  3.9 (2.3 – 62.4) |
| BDE-52  Mean ± SE  Median (10-90 pctl) | 6% | 7.8 ± 0.5  6.6 (3.8 – 12.3) | 7.8 ± 0.7  6.2 (3.6 – 11.6) | 7.7 ± 0.9  7.0 (3.9 – 13.7) |
| BDE-95  Mean ± SE  Median (10-90 pctl) | 48% | 2.3 ± 0.7  0.0 (0.0 – 8.5) | 1.9 ± 0.7  0.0 (0.0 – 5.3) | 3.0 ± 1.3  0.0 (0.0 – 16.4) |
| BDE-99  Mean ± SE  Median (10-90 pctl) | 82% | 3,159 ± 1,117  570 (0.0 – 5,890) | 1,261 ± 385  179 (0.0 – 3,807) | 6,101 ± 2,694  995 (17.8 – 31,908) |
| BDE-100  Mean ± SE  Median (10-90 pctl) | 52% | 107 ± 41.5  0.4 (0.0 – 213) | 21.3 ± 10.7  0.0 (0.0 – 85.2) | 240 ± 98.6  0.5 (0.0 – 1028) |
| BDE-136  Mean ± SE  Median (10-90 pctl) | 12% | 1.5 ± 0.8  0.0 (0.0 – 2.6) | 0.8 ± 0.5  0.0 (0.0 – 0.1) | 2.6 ± 2.0  0.0 (0.0 – 6.7) |
| BDE-153  Mean ± SE  Median (10-90 pctl) | 83% | 228 ± 85  37 (0.0 – 470) | 101.2 ± 35.6  12.6 (0.0 – 269) | 425 ± 204  39.8 (0.2 – 2,080) |
| BDE-154  Mean ± SE  Median (10-90 pctl) | 65% | 176 ± 66.2  24.2 (0.0 – 275) | 71.4 ± 22.5  18.1 (0.0 – 238) | 337 ± 161  45.5 (0.0 – 1,557) |
| BDE-183  Mean ± SE  Median (10-90 pctl) | 13% | 25.2 ± 11.8  0.0 (0.0 – 26.1) | 27.0 ± 14.4  0.0 (0.0 – 120) | 22.5 ± 20.8  0.0 (0.0 – 16.4) |
| ΣPBDEs  Mean ± SE  Median (10-90 pctl) |  | 4,787 ± 1,467  958 (79.5 – 8,758) | 1,972 ± 523  730 (62.4 – 6,363) | 9,151 ± 3,483  2,664 (287 – 40,736) |
| ΣPBDEs > 40%DFR  Mean ± SE  Median (10-90 pctl) |  | 4,692 ± 1,467  812 (38.3 – 8,723) | 1,907 ± 524  692 (36.0 – 6,339) | 9,008 ± 3,487  2632 (255 – 40,701 |
| **PCBs** |  |  |  |  |
| PCB-91  Mean ± SE  Median (10-90 pctl) | 17% | 76.1 ± 36.2  1.8 (1.3 – 284) | 1.9 ± 0.2  1.8 (1.2 – 2.7) | 158 ± 72.6  1.8 (1.4 – 832) |
| PCB-95  Mean ± SE  Median (10-90 pctl) | 17% | 415 ± 202  0.40 (0.2 – 1,566) | 0.4 ± 0.03  0.5 (0.2 – 0.6) | 873 ± 405  0.4 (0.2 – 4,699) |
| PCB-131  Mean ± SE  Median (10-90 pctl) | 44% | 7.4 ± 2.5  0.8 (0.5 – 13.8) | 3.0 ± 0.9  0.8 (0.6 – 5.9) | 14.4 ± 5.9  0.8 (0.5 – 64.6) |
| PCB-135  Mean ± SE  Median (10-90 pctl) | 32% | 73.2 ± 30.1  9.0 (2.0 – 292) | 10.5 ± 2.9  4.6 (1.7 – 21.6) | 142 ± 60.2  10.7 (2.3 – 719) |
| PCB-136  Mean ± SE  Median (10-90 pctl) | 32% | 107 ± 46.9  1.8 (0.9 – 472) | 14.0 ± 6.1  1.9 (0.9 – 46.8) | 210 ± 94.2  1.4 (0.9 – 999) |
| PCB-153  Mean ± SE  Median (10-90 pctl) | 71% | 499 ± 176  78.6 (1.2 – 1,846) | 118 ± 49.1  55.7 (1.0 – 468) | 1,118 ± 370  1,042 (2.2 – 2,370) |
| PCB-174  Mean ± SE  Median (10-90 pctl) | 51% | 93.3 ± 29.3  2.4 (1.0 – 395) | 44.2 ± 15.8  2.8 (1.0 – 151) | 148 ± 58  1.5 (1.1 – 632) |
| PCB-175  Mean ± SE  Median (10-90 pctl) | 37% | 11.7 ± 1.5  9.3 (1.8 – 21.9) | 9.5 ± 1.1  10.4 (1.8 – 16.7) | 15.1 ± 3.4  8.9 (3.4 – 45.0) |
| PCB-176  Mean ± SE  Median (10-90 pctl) | 40% | 37.4 ± 5.2  26.6 (14.6 – 69.2) | 30.7 ± 3.1  27.3 (14.5 – 43.2) | 47.8 ± 12.3  20.8 (14.7 – 156) |
| PCB-180  Mean ± SE  Median (10-90 pctl) | 90% | 351 ± 85.7  175 (26.5 – 909) | 177 ± 50.5  96.9 (1.8 – 481) | 633 ± 172  653 (47.6 – 1,229) |
| PCB-196  Mean ± SE  Median (10-90 pctl) | 48% | 29.3 ± 6.4  3.9 (2.3 – 96.3) | 19.9 ± 5.2  3.9 (2.5 – 60.7) | 44.0 ± 13.7  8.3 (2.2 – 155) |
| ΣPCBs  Mean ± SE  Median (10-90 pctl) |  | 1,036 ± 405  54.5 (36.5 – 1,391) | 235 ± 73.3  58.7 (36.1 – 435) | 2,277 ± 976  54.3 (37.7 – 10,780) |
| ΣPCBs > 40% DFR  Mean ± SE  Median (10-90 pctl) |  | 497 ± 158  34.5 (18.5 – 1,251) | 207 ± 67.1  36.3 (18.3 – 414.3) | 947 ± 372  30.8 (19.0 – 4,114) |

**Table S2.** Odds ratios^a^ of feline hyperthyroidism by serum concentrations of the four most abundant PBDE and PCB congeners^b^.

| **BDE or PCB Congener** | **Odds Ratio^a^ (95% CI)** | **p-value** |
| --- | --- | --- |
| PBDEs |  |  |
| BDE 47 | 1.06 (1.00 – 1.13) | 0.030***** |
| BDE 99 | 1.01 (1.00 – 1.02) | 0.230 |
| PCBs |  |  |
| PCB 153 | 1.29 (1.04 – 2.13) | 0.010***** |
| PCB 180 | 1.48 (1.06 – 2.42) | 0.014***** |

^a^ Odds ratios represent the increase in odds associated with a 100 ng/g lipid increase in congener concentration.

^b^ The logistic regression analyses of the relationship between serum concentrations of PBDE and PCB congeners and feline hyperthyroidism controls for age of feline study participants.

* p-value ≤ 0.050

**Table S3.** Odds ratios^a^ of feline hyperthyroidism by serum concentrations of PBDE and PCB congeners^b^.

| **BDE or PCB Congener** | **Odds Ratio (95% CI)** | **p-value** |
| --- | --- | --- |
| PBDEs |  |  |
| BDE-17 | 1.83 (1.06 – 2.95) | 0.021***** |
| BDE-28 | 1.31 (0.54 – 3.16) | 0.549 |
| BDE-38 | 1.16 (0.84 – 1.61) | 0.377 |
| BDE-49 | 1.72 (1.07 – 3.02) | 0.021***** |
| BDE-52 | 1.20 (0.22 – 6.62) | 0.831 |
| BDE-95 | 2.18 (0.56 – 8.53) | 0.263 |
| BDE-100 | 1.09 (1.01 – 1.19) | 0.008***** |
| BDE-136 | 2.14 (0.57 – 7.97) | 0.258 |
| BDE-153 | 1.01 (0.99 – 1.02) | 0.274 |
| BDE-154 | 1.01 (0.99 – 1.04) | 0.263 |
| BDE-183 | 1.02 (0.95 – 1.11) | 0.576 |
| PCBs |  |  |
| PCB-91 | 1.14 (0.54 – 2.39) | 0.731 |
| PCB-95 | 1.03 (0.66 – 1.61) | 0.892 |
| PCB-131 | 1.77 (1.08 – 3.68) | 0.017***** |
| PCB-135 | 1.14 (0.79 – 1.63) | 0.491 |
| PCB-136 | 1.06 (0.97 – 1.15) | 0.242 |
| PCB-174 | 1.05 (1.00 – 1.11) | 0.042***** |
| PCB-175 | 1.74 (0.99 – 3.44) | 0.055 |
| PCB-176 | 1.17 (0.99 – 1.43) | 0.071 |
| PCB-196 | 1.17 (1.02 – 1.38) | 0.029***** |

^a^ Odds ratios represent the increase in odds associated with a 10 ng/g lipid increase in congener concentration.

^b^ The logistic regression analyses of the relationship between serum concentrations of PBDE and PCB congeners and feline hyperthyroidism controls for age of feline study participants.

* p-value ≤ 0.050
